# Distributed adaptive fixed-time neural networks control for nonaffine nonlinear multiagent systems

**DOI:** 10.1038/s41598-022-12634-2

**Published:** 2022-05-19

**Authors:** Yang Li, Quanmin Zhu, Jianhua Zhang

**Affiliations:** 1 School of Information and Control Engineering, Qingdao University of Technology, Qingdao, 266525 China; 2 Department of Engineering Design and Mathematics, University of the West of England, Coldharbour Lane, Bristol, BS16 1QY UK

**Keywords:** Mechanical engineering, Engineering

## Abstract

This paper, with the adaptive backstepping technique, presents a novel fixed-time neural networks leader–follower consensus tracking control scheme for a class of nonaffine nonlinear multiagent systems. The expression of the error system is derived, based on homeomorphism mapping theory, to formulate a set of distributed adaptive backstepping neural networks controllers. The weights of the neural networks controllers are trained, by an adaptive law based on fixed-time theory, to determine the adaptive control input. The control algorithm can guarantee that the output of the follower agents of the system effectively follow the output of the leader of the system in a fixed time, while the upper bound of the settling time can be calculated without initial parameters. Finally, a simulation example is presented to demonstrate the effectiveness of the proposed consensus tracking control approach. A step-by-step procedure for engineers and researchers interested in applications is proposed.

## Introduction

In practical engineering, many control systems are modelled by nonlinear dynamics, such as inverted pendulum mechanical systems^1,2^. Because it is difficult to solve nonlinear mathematical equations, there is no unified methodology of studying different types of nonlinear control systems due to the loss of superposition principle. For a system with mild nonlinearity, the linearization-based control method has been widely used^2–4^. For systems with inherent nonlinearities, such as single-input single-output systems, nonaffine nonlinear systems, triangular nonlinear systems, high-order nonlinear systems^5–7^, and multiagent nonlinear systems^8–10^, various remarkable studies have been devoted to the system analysis and control design^11,12^.

With the improvement of industrial technology, the actual control systems faced by engineers are becoming increasingly complex, and the mathematical models of these systems are increasingly complicated with functionality and structure, which is because the performance request of the modern engineering systems/products is increasingly higher to satisfy the human being’s ever increased demands/expectations. Consequently, using these corresponding control theories require extremely profound mathematical foundation. There is a certain gap between control theory and practical control engineering. It is necessary to study to bridge control theory and its applications, such great efforts have been widely witnessed^13–15^. At present, there are two predominant methodologies used to solve this problem. The first methodology is the computational thinking based artificial intelligence technology, such as computer vision^16^, language processing^17^ and pattern recognition^18^. The second methodology is the control theory based nonlinear control technology, such as chaotic synchronization^19^, multiagent consensus, nonlinear tracking control^20^, robot control^21^, and unmanned aerial vehicle control^22^. The two types of methodologies have been intensively/extensively adopted to support multiagent systems in academia research and applications^23–25^.

In engineering practice, most systems often have control objectives within a limited convergence time, such as missile systems, because missiles do not need control after explosion. Regarding the finite-time stability^26^ and stabilization of the controlled system, the convergence time can be determined accurately, which is important in applications. However, the approach induces difficulties in applications due to the bound of the convergence time in the control systems is always related with initial states and control gain^27–29^. To cope with the problem of initial state dependent boundness, the fixed-time stability and stabilization approach has been developed^30,31^, so that the controlled system is stabilized in finite time and the upper bound of the settling time is met by only adjusting the parameters of the controller^32^. As always, every approach has two side effects. The disadvantage of fixed-time control is that the controller is relatively complicated, especially for high order nonlinear systems^33^, the controller singularity problem could arise in the design of the backstepping iterative controller^34–36^. Even the challenging issues in math and system analysis and design, the importance of the fixed-time requests to the dynamic systems have still actively promoted various top journal publications^37,38^ recently. In short, the field of the research is relatively new, need wider angle of studies for understanding and solutions.

For the interest of the studying problems—control of multiagent systems in short, in recent years, there have been many published papers, name a few for reference, concerning the fixed-time control and analysis of closed loop control systems^39–41^, finite-time and fixed-time synchronization control for complex network systems with distributed protocols^42^, finite-time and fixed-time stability analysis for a class of high-order neural networks with delays based on the linear inequality matrix technique^43^, fixed-time event leader–follower event-triggered consensus control for multiple agents, fixed-time tracking control for second-order multi-agent system with bounded input uncertainties is studied in^44^, fixed-time consensus framework^45^, observer based distributed fixed-time consensus control for nonlinear leader–follower multi-agent systems^46^, distributed adaptive neural networks consensus tracking control for non-affine nonlinear multi-agent systems is studied in^47^.

What more can the study contribute? several recently published authoritative works, related to distributed adaptive neural networks consensus for a class of uncertain nonaffine nonlinear multi-agent systems^9,47,48^, are selected to compare to justify the contribution.

In papers^9,47,48^, all the closed-loop signals are locally uniformly bounded, and all the subsystem outputs asymptotically stable, therefore, the system is asymptotically stable, the outputs converge exponentially which means stability in infinite time. In the new study, fixed-time control is proposed to design the upper bound of the convergence time of the controlled system. Based on fixed-time control, the bound of convergence time independent from the initial conditions of the system.

In papers^9,47,48^, the adaptive law is designed to training neural networks weights, based on Lyapunov stability theory, estimated weights convergence to ideal weights infinite time. Once again, this study presents a fixed-time adaptive law to training the neural networks weights, which makes the parameters of neural networks iteratively updated in fixed time. It is proved that the bound of convergence time between estimated weights and ideal weights are independent from the initial conditions of the estimated weights.

In papers^9,47^, the states of system are not restricted in process. This study presents homeomorphism mapping technology to make a multiagent system transform to ensure steady-state and transient performance. Combining with the homeomorphism mapping technology and fixed-time, the designed adaptive fixed-time control has guaranteed that all the closed-loop signals are bounded, the system state tracking errors can remain within the predesigned performance regions with fixed-time convergence rate.

The rest of the study consists of the following sections: Section "Problem formulation and preliminaries" presents a mathematical description of the problem as the foundation for providing solutions. Section "Main results" establishes a platform for the distributed adaptive fixed-time neural networks control for nonaffine nonlinear leader–follower multiagent system consensus. Section "Simulation study" validates the performance of the consensus tracking algorithm by a simulated example and further the computational procedure could be a transparent user guide for future expansions and applications. Section "Conclusions" concludes the study.

## Problem formulation and preliminaries

Consider a class of leader–follower multiagent nonaffine nonlinear systems that have a leader 0 and followers $$N$$$$\left( {N \ge 2} \right)$$. The $$i\;{\text{th}}$$ follower agent of the nonaffine nonlinear multiagent system model is given by

1 $$\begin{aligned} \dot{x}_{i,m} & = f_{i,m} \left( {\overline{x}_{i,m} ,x_{i,m + 1} } \right),\quad m = 1, \ldots ,n_{i} - 1 \\ \dot{x}_{{i,n_{i} }} & = f_{{i,n_{i} }} \left( {\overline{x}_{{i,n_{i} }} ,u_{i} } \right) \\ y_{i} & = x_{i,1} \\ \end{aligned}$$

### Assumption 1

The sign of $\frac{{\partial f_{i,m} \left( {\overline{x}_{i,m} ,x_{i,m + 1} } \right)}}{{\partial x_{i,m + 1} }}$ is assumed to be either strictly positive or strictly negative in most articles, and we assume that $\frac{{\partial f_{i,m} \left( {\overline{x}_{i,m} ,x_{i,m + 1} } \right)}}{{\partial x_{i,m + 1} }} > 0$ in this article, where $m = 1,2, \cdots ,n_{i}$ and $x_{{i,n_{i} + 1}} = u_{i}$.

The follower agent system function can be described as follows based on the mean value theorem:

2 $$\begin{aligned} \dot{x}_{i,m} & = f_{i,m} \left( {\overline{x}_{i,m} ,0} \right) + g_{i,m} \left( {\overline{x}_{i,m} ,x_{i,m + 1}^{0} } \right)x_{i,m + 1} \\ \dot{x}_{{i,n_{i} }} & = f_{{i,n_{i} }} \left( {\overline{x}_{{i,n_{i} }} ,0} \right) + g_{{i,n_{i} }} \left( {\overline{x}_{{i,n_{i} }} ,u_{i1}^{0} } \right)u_{i,} \\ y_{i} & = x_{i,1} \\ \end{aligned}$$

where $0 < \lambda_{i,m + 1} < 1$ and $u_{i}^{0} = \lambda_{{i,n_{i} }} u_{i}$, with $0 < \lambda_{{i,n_{i} }} < 1$, where $x_{i,m} \in {\mathbf{\mathbb{R}}}$ is the $m\;{\text{th}}$ state of the nonaffine nonlinear multiagent system $i$, $\overline{x}_{i,m} = \left[ {x_{i,1} , \ldots ,x_{i,m} } \right]^{T} \in {\mathbf{\mathbb{R}}}^{m}$ is the state vector of the system, $y_{i} \in {\mathbf{\mathbb{R}}}$ is the output of the system, $u_{i} \in {\mathbf{\mathbb{R}}}$ indicates the controller that needs to be designed, and $f_{i,m} \left( {\overline{x}_{i,m} ,x_{i,m + 1} } \right):{\mathbf{\mathbb{R}}}^{m + 1} \to {\mathbf{\mathbb{R}}}$ is the unknown smooth nonlinear function.

### Graph theory

Assume $G = \left( {V,E} \right)$ is a directed graph, $E \subseteq V \times V$ is the edge set, and $V = \left\{ {v_{1} ,v_{2} , \cdots ,v_{N} } \right\}$ is the node set. An edge $e_{ji} = \left( {v_{j} ,v_{i} } \right) \in E$ of graph $G$ indicates that $i$ can get messages from $j$, where agent $j$ is one of agent $i$’s neighbours.

The node set indicates communication among agents. Hence, agent $i$’s neighbour set is $N_{i} = \left\{ {v_{j} \left| {\left( {v_{j} ,v_{i} } \right) \in E} \right.} \right\}$. The directed graph is called a weighted graph when the edges have weights $A = \left[ {a_{ij} } \right] \in {\mathbf{\mathbb{R}}}^{N \times N}$ (adjacency matrix), and such graphs are often used to express the graphical topology. For element $a_{ij}$, it is defined that $a_{ij} = 1$ if $e_{ji} = \left( {v_{j} ,v_{i} } \right) \in E$; otherwise, $a_{ij} = 0$. The self-loop is not considered, as usual, i.e., $a_{ii} = 0$, and the degree matrix is denoted as $D = diag\left( {d_{1} ,d_{2} , \cdots d_{N} } \right) \in {\mathbf{\mathbb{R}}}^{N \times N}$ with $d_{i} = \sum\limits_{j = 1}^{k} {a_{ij} }$.

For a consensus error $\delta$, where $\delta { = }\left[ {\delta_{1} , \ldots ,\delta_{N} } \right]^{T}$ with $\delta_{i} = y_{i} - y_{0} ,i = 1, \ldots ,N$, consider a directed graph as $\left\{ {\left( {v_{i} ,v_{r} } \right),\left( {v_{r} ,v_{s} } \right), \cdots ,\left( {v_{t} ,v_{j} } \right)} \right\}$. The local tracking error for agent $i$ can be described as

3 $$\xi_{i,1} = \sum\limits_{j = 1}^{{N_{i} }} {a_{ij} \left( {y_{i} - y_{j} } \right) + b_{i} } \left( {y_{i} - y_{0} } \right),$$

which can be measured distributively^48^. Suppose $\underline {\xi }_{i,1} \left( t \right)$ and $\overline{\xi }_{i,1} \left( t \right)$ are known bounds of $\xi_{i,1}$. To ensure the constraint control of system nonlinear homeomorphism mapping^26^, system (2) is transformed as follows:

4 $$\xi = \frac{2a}{\pi }\arctan \left( z \right),\xi = a\tanh \left( z \right),\xi = {\text{sgn}} \left( x \right)a\left( {1 - e^{{ - z^{2} }} } \right)^{\frac{1}{2}} ,$$

where $a = \max \left( {\underline {\xi }_{i,1} \left( t \right),\overline{\xi }_{i,1} \left( t \right)} \right)$, and we assume that

5 $$r = \frac{\partial z}{{\partial \xi }}.$$

### RBF (radial basis function) neural networks

RBF neural networks have a strong approximation ability for nonlinear functions, such as

6 $$F\left( x \right) = W^{T} \Psi \left( x \right) + \varepsilon \left( x \right),$$

where $W$ is the ideal weight of the neural networks and $\varepsilon \left( x \right)$ is the neural networks approximation error as follows:

7 $$W = \arg \mathop {\min }\limits_{{W \in \Re^{l} }} \left\{ {\sup \left| {F\left( x \right) - W^{T} \Psi \left( x \right)} \right|} \right\}.$$

#### **Notation**

 In this article, $W$ represents the ideal weight, and $\hat{W}$ represents the estimated weight. Then, $\theta_{i,j} = \left\| {W_{i,j} } \right\|$,$\hat{\theta }_{i,j} = \left\| {\hat{W}_{i,j} } \right\|$ and $\tilde{\theta }_{i,j} = \hat{\theta }_{i,j} - \theta_{i,j}$ hold.

#### Remark 1

Exponential stability, finite time stability, and fixed-time stability are well known. For example, the system $\dot{x} = - x$ is exponentially stable, the system $\dot{x} = - x^{\frac{1}{3}}$ is finite-time stable, and the system $\dot{x} = - x^{\frac{1}{3}} - x^{3}$ is fixed-time stable (see^49^ for details). In the next section, Theorem 1 provides the adaptive fixed-time neural networks tracking control scheme, which implies the existence of the Lyapunov function, and fixed-time stability is also proven.

In the next section, the distributed adaptive fixed-time neural networks controller is designed based on fixed-time stability theory. The control objective is for the follower agents to be able to track the leader agent in fixed time and maintain fixed-time stability based on the distributed adaptive fixed-time neural networks controller. The upper bound of settling time can be designed without the initial parameters.

## Main results

### Distributed adaptive fixed-time neural networks consensus control scheme

In this section, the distributed adaptive design approach incorporates fixed-time stability theory, and the distributed adaptive neural networks controller based on the backstepping technique is designed for a class of nonaffine nonlinear leader–follower multiagent systems. Neural networks are designed to approximate the unknown parameters. Adaptive fixed-time laws are designed to train the weights of the neural networks. Based on the controller, the error closed system achieves fixed-time consensus, which means that the follower agent can track the leader agent in fixed time.

#### Remark 2

**Figure 1:**
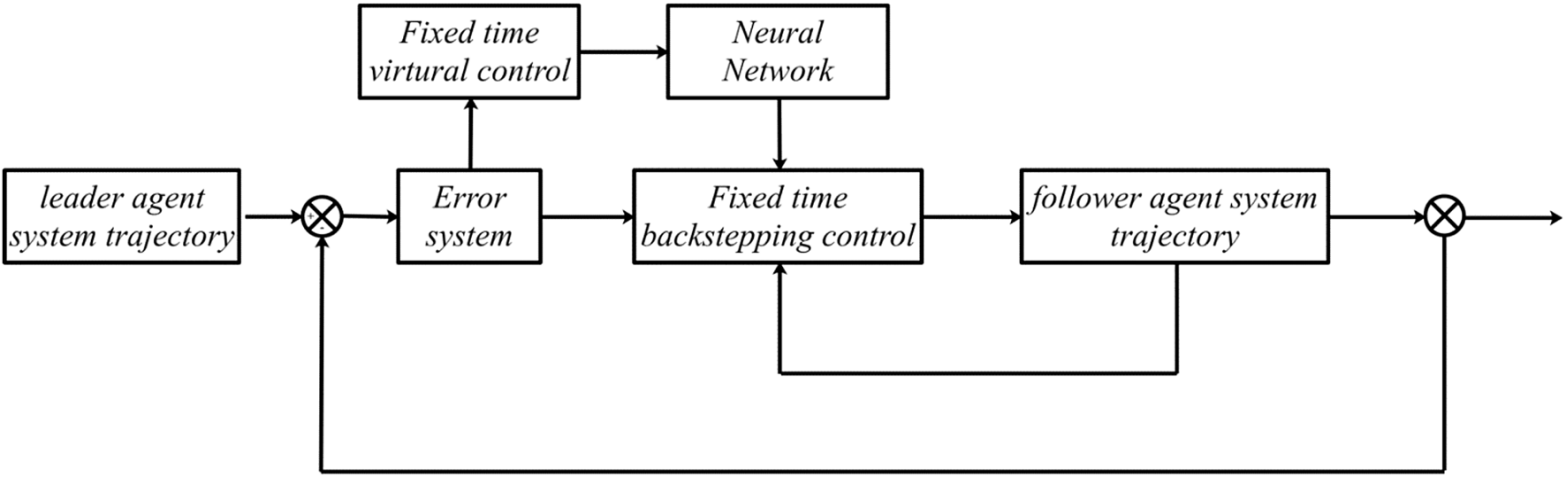
Consensus control structure diagram of the closed system.

The control structure block diagram for the nonaffine nonlinear leader–follower multiagent system is shown in Fig. 1. The consensus control scheme structure of the closed-loop system is shown in Fig. 1. The consensus control objective is that the output of the follower agent can track the leader agent signal. In the next section, the stability analysis and mathematical proof based on the fixed-time consensus theorem will be given.

### Fixed-time stabilization based on distributed adaptive fixed-time neural networks consensus control

Based on the dynamics and local tracking error of the $i\;{\text{th}}$ follower agent (1), local tracking error of the $i\;{\text{th}}$ follower agent (3) and homeomorphism mapping (4), the dynamics of $z_{i,1} ,i = 1, \ldots ,N$ can be obtained as

8 $$\dot{z}_{i,1} = r_{i} \left[ {\left( {d_{i} + b_{i} } \right)\dot{y}_{i} - \sum\limits_{{j \in N_{i} }} {a_{ij} \dot{y}_{j} } - b_{i} \dot{y}_{0} } \right]$$

and $r_{i} = \frac{{\partial z_{i,1} }}{{\partial \xi_{i,1} }}$; based on system (2), we have

9 $$\dot{z}_{i,1} = r_{i} \left( {d_{i} + b_{i} } \right)F_{i,1} \left( {X_{i,1} } \right) + r_{i} \left( {d_{i} + b_{i} } \right)g_{i,1} x_{i,2} - r_{i} b_{i} \dot{y}_{0} ,$$

where

10 $$F_{i,1} \left( {X_{i,1} } \right) = f_{i,1} - \frac{1}{{\left( {d_{i} + b_{i} } \right)}}\sum\limits_{{j \in N_{i} }} {a_{ij} \left( {f_{j,1} + g_{j,1} x_{j,2} } \right)} .$$

Moreover, the neural networks approximate the nonlinear system

11 $$F_{i,1} \left( {X_{i,1} } \right) = W_{i,1}^{T} \Psi \left( {Z^{\prime}_{i,1} } \right) + \varepsilon_{i,1} \left( {Z^{\prime}_{i,1} } \right).$$

For the neural networks approximation error, assuming that $\left| {\varepsilon_{i,1} \left( {Z^{\prime}_{i,1} } \right)} \right| \le \overline{\varepsilon }_{i,1}$, based on the Lemma in^48^, the following inequality can be obtained:

12 $$F_{i,1} \left( {X_{i,1} } \right) \le \left\| {W_{i,1} } \right\|\left\| {\Psi \left( {Z_{i,1} } \right)} \right\| + \overline{\varepsilon }_{i,1} .$$

The virtual control $\alpha_{i,1}$ is designed as

13 $$\begin{aligned} \alpha_{i,1} & = - \frac{{k_{p,i,1} z_{i,1}^{p} }}{{\underline {g}_{i,1} \left( {d_{i} + b_{i} } \right)r_{i} }} - \frac{{k_{q,i,1} z_{i,1}^{q} }}{{\underline {g}_{i,1} \left( {d_{i} + b_{i} } \right)r_{i} }} - \frac{{r_{i} z_{i,1} \hat{\theta }_{i,1}^{2} \left\| {\Psi \left( {Z_{i,1} } \right)} \right\|^{2} }}{{\underline {g}_{i,1} \left( {\left| {r_{i} z_{i,1} \hat{\theta }_{i,1} } \right|\left\| {\Psi \left( {Z_{i,1} } \right)} \right\| + \eta_{1,i,1} } \right)}} \\ & \quad - \frac{{r_{i} z_{i,1} \overline{\varepsilon }_{i,1}^{2} }}{{\underline {g}_{i,1} \left( {\left| {r_{i} z_{i,1} } \right|\overline{\varepsilon }_{i,1} + \eta_{2,i,1} } \right)}} - \frac{{r_{i} z_{i,1} b_{i}^{2} \dot{y}_{0}^{2} }}{{\underline {g}_{i,1} \left( {d_{i} + b_{i} } \right)\left( {\left| {z_{i,1} r_{i} b_{i} \dot{y}_{0} } \right| + \eta_{3,i,1} } \right)}} \\ \end{aligned}$$

where

14 $$z_{i,2} = x_{i,2} - \alpha_{i,1} .$$

Taking the derivative of $z_{i,m} ,2 \le m \le n$ yields

15 $$\dot{z}_{i,m} = f_{i,m} + g_{i,2} x_{i,m + 1} - \dot{\alpha }_{i,m - 1} .$$

Then,

16 $$F_{i,m} \left( {X_{i,m} } \right) = f_{i,m} - \dot{\alpha }_{i,m - 1} + z_{i,m - 1} g_{i,m - 1} .$$

Moreover, the following equation can be obtained:

17 $$F_{i,m} \left( {X_{i,m} } \right) = W_{i,m}^{T} \Psi \left( {Z^{\prime}_{i,m} } \right) + \varepsilon_{i,m} \left( {Z^{\prime}_{i,m} } \right).$$

For the neural networks approximation error, assuming that $\left| {\varepsilon_{i,m} \left( {Z^{\prime}_{i,m} } \right)} \right| \le \overline{\varepsilon }_{i,m}$, based on the lemma in^48^, the following inequality can be obtained:

18 $$F_{i,m} \left( {X_{i,m} } \right) \le \left\| {W_{i,m} } \right\|\left\| {\Psi \left( {Z_{i,m} } \right)} \right\| + \overline{\varepsilon }_{i,m} .$$

The virtual control $\alpha_{i,m}$ is designed as

19 $$\alpha_{i,m} = - \frac{1}{{\underline {g}_{i,m} }}\left( {k_{p,i,m} z_{i,m}^{p} + k_{q,i,m} z_{i,m}^{q} + \frac{{z_{i,m} \hat{\theta }_{i,m}^{2} \left\| {\Psi \left( {Z_{i,m} } \right)} \right\|^{2} }}{{\left| {z_{i,m} \hat{\theta }_{i,m} } \right|\left\| {\Psi \left( {Z_{i,m} } \right)} \right\| + \eta_{1,i,m} }} + \frac{{z_{i,2} \overline{\varepsilon }_{i,m}^{2} }}{{\left| {z_{i,m} } \right|\overline{\varepsilon }_{i,m} + \eta_{2,i,m} }}} \right),$$

where

20 $$z_{i,m + 1} = x_{i,m + 1} - \alpha_{i,m} ,$$

and the control is designed as $u_{i} = \alpha_{i,n}$, where

21 $$u_{i} = - \frac{1}{{\underline {g}_{i,n} }}\left( {k_{p,i,n} z_{i,n}^{p} + k_{q,i,n} z_{i,n}^{q} + \frac{{z_{i,n} \hat{\theta }_{i,n}^{2} \left\| {\Psi \left( {Z_{i,n} } \right)} \right\|^{2} }}{{\left| {z_{i,n} \hat{\theta }_{i,n} } \right|\left\| {\Psi \left( {Z_{i,n} } \right)} \right\| + \eta_{1,i,n} }} + \frac{{z_{i,2} \overline{\varepsilon }_{i,n}^{2} }}{{\left| {z_{i,n} } \right|\overline{\varepsilon }_{i,n} + \eta_{2,i,n} }}} \right).$$

The neural networks adaptive law is designed as

22 $$\dot{\hat{\theta }}_{i,j} = \mu_{i,j} \left( {\left| {z_{i,j} } \right|\left\| {\Psi \left( {Z_{i,j} } \right)} \right\| - \rho_{i,j} \hat{\theta }_{i,j}^{p} - \sigma_{i,j} \hat{\theta }_{i,j}^{q} } \right),1 \le i \le N,1 \le j \le n.$$

#### Theorem 1

Consider the nonaffine nonlinear leader–follower multiagent system (1) and local tracking error system (3); based on the adaptive fixed-time neural networks control scheme and backstepping technique, choose the virtual control law as (13) and (19), the distributed adaptive fixed-time law as (22), and the actual controller as (21). The tracking error system is a fixed-time consensus, and the upper bound of the settling time $T$ is independent from the initial parameters. The settling time $T$ satisfies.

23 $$T \le T_{\max } = \frac{{2^{{\frac{3 - p}{2}}} }}{{k_{p} \left( {1 - p} \right)}} + \frac{2}{{k_{q} \left( {q - 1} \right)}}.$$

#### *Proof*

 Choose the Lyapunov candidate functional as

24 $$V_{i,1} = \frac{1}{2}z_{i,1}^{2} + \frac{1}{{2\mu_{i,1} }}\tilde{\theta }_{i,1}^{2} ,$$

where $\mu_{i,1} > 0$ is a positive constant. Differentiating $V_{i,1}$ with respect to time $t$ yields

25 $$\begin{aligned} \dot{V}_{i,1} & = z_{i,1} r_{i} \left( {d_{i} + b_{i} } \right)F_{i,1} \left( {X_{i,1} } \right) + z_{i,1} r_{i} \left( {d_{i} + b_{i} } \right)g_{i,1} \left( {x_{i,1} ,x_{i,2}^{0} } \right)x_{i,2} \\ & \quad - z_{i,1} r_{i} b_{i} \dot{y}_{0} + \frac{1}{{\mu_{i,1} }}\tilde{\theta }_{i,1} \dot{\hat{\theta }}_{i,1} \\ \end{aligned}$$

Then, the following inequality can be obtained:

26 $$\begin{aligned} \dot{V}_{i,1} & \le \left( {d_{i} + b_{i} } \right)\left( {\left| {r_{i} z_{i,1} } \right|\left\| {W_{i,1} } \right\|\left\| {\Psi \left( {Z_{i,1} } \right)} \right\| - \frac{{r_{i}^{2} z_{i,1}^{2} \hat{\theta }_{i,1}^{2} \left\| {\Psi \left( {Z_{i,1} } \right)} \right\|^{2} }}{{\left| {r_{i} z_{i,1} \hat{\theta }_{i,1} } \right|\left\| {\Psi \left( {Z_{i,1} } \right)} \right\| + \eta_{1,i,1} }}} \right) \\ & \quad + \left( {d_{i} + b_{i} } \right)\left( {\left| {r_{i} z_{i,1} } \right|\overline{\varepsilon }_{i,1} - \frac{{r_{i}^{2} z_{i,1}^{2} \overline{\varepsilon }_{i,1}^{2} }}{{\left| {r_{i} z_{i,1} } \right|\overline{\varepsilon }_{i,1} + \eta_{2,i,1} }}} \right) \\ & \quad + \left| {z_{i,1} r_{i} b_{i} \dot{y}_{0} } \right| - \frac{{r_{i}^{2} z_{i,1}^{2} b_{i}^{2} \dot{y}_{0}^{2} }}{{\left| {z_{i,1} r_{i} b_{i} \dot{y}_{0} } \right| + \eta_{3,i,1} }} \\ & \quad - \frac{{z_{i,1} r_{i} \left( {d_{i} + b_{i} } \right)g_{i,1} }}{{\underline {g}_{i,1} }}\left( {\frac{{k_{p,i,1} z_{i,1}^{p} }}{{r_{i} \left( {d_{i} + b_{i} } \right)}} + \frac{{k_{q,i,1} z_{i,1}^{q} }}{{r_{i} \left( {d_{i} + b_{i} } \right)}}} \right) \\ & \quad + \frac{1}{{\mu_{i,1} }}\tilde{\theta }_{i,1} \dot{\hat{\theta }}_{i,1} + z_{i,1} r_{i} \left( {d_{i} + b_{i} } \right)g_{i,1} z_{i,2} \\ \end{aligned}$$

Based on the lemma in^48^ and the inequality technique, it follows that

27 $$\begin{aligned} \dot{V}_{i,1} & \le - \left( {d_{i} + b_{i} } \right)\left| {r_{i} z_{i,1} } \right|\left\| {\Psi \left( {Z_{i,1} } \right)} \right\|\tilde{\theta }_{i,1} \\ & \quad + \left( {d_{i} + b_{i} } \right)\left( {\eta_{1,i,1} + \eta_{2,i,1} } \right) + \eta_{3,i,1} - k_{p,i,1} z_{i,1}^{p + 1} - k_{q,i,1} z_{i,1}^{q + 1} \\ & \quad + \frac{1}{{\mu_{i,1} }}\tilde{\theta }_{i,1} \dot{\hat{\theta }}_{i,1} + z_{i,1} r_{i} \left( {d_{i} + b_{i} } \right)g_{i,1} z_{i,2} \\ \end{aligned}$$

The actual controller is designed as

28 $$u_{i} = - \frac{1}{{\underline {g}_{i,n} }}\left( {k_{p,i,n} z_{i,n}^{p} + k_{q,i,n} z_{i,n}^{q} + \frac{{z_{i,n} \hat{\theta }_{i,n}^{2} \left\| {\Psi \left( {Z_{i,n} } \right)} \right\|^{2} }}{{\left| {z_{i,n} \hat{\theta }_{i,n} } \right|\left\| {\Psi \left( {Z_{i,n} } \right)} \right\| + \eta_{1,i,n} }} + \frac{{z_{i,2} \overline{\varepsilon }_{i,n}^{2} }}{{\left| {z_{i,n} } \right|\overline{\varepsilon }_{i,n} + \eta_{2,i,n} }}} \right).$$

Based on the adaptive law (22) of the controller (28) and the lemma in^32^, the following inequality can be obtained:

29 $$\begin{aligned} \dot{V}_{i,1} & \le - k_{p,i,1} z_{i,1}^{p + 1} - k_{q,i,1} z_{i,1}^{q + 1} - \rho_{i,1} \tilde{\theta }_{i,1} \hat{\theta }_{i,1}^{p} - \sigma_{i,1} \tilde{\theta }_{i,1} \hat{\theta }_{i,1}^{q} \\ & \quad + \left( {d_{i} + b_{i} } \right)\eta_{1,i,1} + \left( {d_{i} + b_{i} } \right)\eta_{2,i,1} + \eta_{3,i,1} \\ & \quad + z_{i,1} r_{i} \left( {d_{i} + b_{i} } \right)g_{i,1} z_{i,2} \\ & \le - k_{p,i,1} z_{i,1}^{p + 1} - k_{q,i,1} z_{i,1}^{q + 1} - \rho_{1,i,1} \tilde{\theta }_{i,1}^{p + 1} - \sigma_{1,i,1} \tilde{\theta }_{i,1}^{q + 1} \\ & \quad + \rho_{2,i,1} \theta_{i,1}^{p + 1} + \sigma_{2,i,1} \theta_{i,1}^{q + 1} + \left( {d_{i} + b_{i} } \right)\eta_{1,i,1} + \left( {d_{i} + b_{i} } \right)\eta_{2,i,1} + \eta_{3,i,1} \\ & \quad + z_{i,1} r_{i} \left( {d_{i} + b_{i} } \right)g_{i,1} z_{i,2} \\ & \le \Sigma_{i,1} + \Delta_{i,1} + z_{i,1} r_{i} \left( {d_{i} + b_{i} } \right)g_{i,1} z_{i,2} \\ \end{aligned}$$

where

30 $$\begin{aligned} \Sigma_{i,1} & = - k_{p,i,1} z_{i,1}^{p + 1} - k_{q,i,1} z_{i,1}^{q + 1} - \rho_{1,i,1} \tilde{\theta }_{i,1}^{p + 1} - \sigma_{1,i,1} \tilde{\theta }_{i,1}^{q + 1} \\ \Delta_{i,1} & = \rho_{2,i,1} \theta_{i,1}^{p + 1} + \sigma_{2,i,1} \theta_{i,1}^{q + 1} + \left( {d_{i} + b_{i} } \right)\eta_{1,i,1} + \left( {d_{i} + b_{i} } \right)\eta_{2,i,1} + \eta_{3,i,1} . \\ \end{aligned}$$

We choose the Lyapunov candidate functional as

31 $$V_{i,m} = V_{i,m - 1} + \frac{1}{2}z_{i,m}^{2} + \frac{1}{{2\mu_{i,m} }}\tilde{\theta }_{i,m}^{2} ,$$

where $\mu_{i,m} > 0$ is a positive constant

32 $$\begin{aligned} \dot{V}_{i,m} & \le \Sigma_{i,m - 1} + \Delta_{i,m - 1} + z_{i,m - 1} g_{i,m - 1} z_{i,m} \\ & \quad + z_{i,m} f_{i,m} + z_{i,m} g_{i,m} x_{i,m + 1} + \frac{1}{{\mu_{i,m} }}\tilde{\theta }_{i,m} \dot{\hat{\theta }}_{i,m} \\ & \le \Sigma_{i,m - 1} + \Delta_{i,m - 1} - \frac{{g_{i,m} }}{{\underline {g}_{i,m} }}\left( {k_{p,i,m} z_{i,m}^{p + 1} + k_{q,i,m} z_{i,m}^{q + 1} } \right) \\ & \quad + \left| {z_{i,m} } \right|\left\| {\Psi \left( {Z_{i,m} } \right)} \right\|\left( {\left\| {W_{i,m} } \right\| - \hat{\theta }_{i,m} } \right) + \left| {z_{i,m} } \right|\left\| {\Psi \left( {Z_{i,m} } \right)} \right\|\hat{\theta }_{i,m} \\ & \quad - \frac{{g_{i,m} z_{i,m}^{2} \hat{\theta }_{i,m}^{2} \left\| {\Psi \left( {Z_{i,m} } \right)} \right\|^{2} }}{{\underline {g}_{i,m} \left( {\left| {z_{i,m} \hat{\theta }_{i,m} } \right|\left\| {\Psi \left( {Z_{i,m} } \right)} \right\| + \eta_{1,i,m} } \right)}} + \left| {z_{i,m} } \right|\overline{\varepsilon }_{i,m} \\ & \quad - \frac{{g_{i,m} }}{{\underline {g}_{i,m} }}\frac{{z_{i,m}^{2} \overline{\varepsilon }_{i,m}^{2} }}{{\left| {z_{i,m} } \right|\overline{\varepsilon }_{i,m} + \eta_{2,i,m + 1} }} + z_{i,m} g_{i,m} z_{i,m + 1} + \frac{1}{{\mu_{i,m} }}\tilde{\theta }_{i,m} \dot{\hat{\theta }}_{i,m} \\ \end{aligned}$$

Based on the lemma in^48^, the following inequality can be obtained:

33 $$\begin{aligned} \dot{V}_{i,m} & \le \Sigma_{i,m - 1} + \Delta_{i,m - 1} - k_{p,i,m} z_{i,m}^{p + 1} - k_{q,i,m} z_{i,m}^{q + 1} \\ & \quad - \left| {z_{i,m} } \right|\left\| {\Psi \left( {Z_{i,m} } \right)} \right\|\tilde{\theta }_{i,m} + \eta_{1,i,m} + \eta_{2,i,m} \\ & \quad + z_{i,m} g_{i,m} z_{i,m} + \frac{1}{{\mu_{i,m} }}\tilde{\theta }_{i,m} \dot{\hat{\theta }}_{i,m} \\ \end{aligned}$$

Based on the adaptive law design, the following inequality can be obtained:

34 $$\begin{aligned} \dot{V}_{i,m} & \le \Sigma_{i,m - 1} + \Delta_{i,m - 1} + \eta_{1,i,m} + \eta_{2,i,m} \\ & \quad - k_{p,i,m} z_{i,m}^{p + 1} - k_{q,i,m} z_{i,m}^{q + 1} + z_{i,m} g_{i,m} z_{i,m} - \rho_{i,m} \tilde{\theta }_{i,m} \hat{\theta }_{i,m}^{p} - \sigma_{i,m} \tilde{\theta }_{i,m} \hat{\theta }_{i,m}^{q} \\ \end{aligned}$$

Based on the lemma in^32^, the following inequality can be obtained:

35 $$\begin{aligned} \dot{V}_{i,m} & \le \Sigma_{i,m - 1} - \rho_{p,i,m} \tilde{\theta }_{i,m}^{p + 1} - \rho_{q,i,m} \tilde{\theta }_{i,m}^{q + 1} - k_{p,i,m} z_{i,m}^{p + 1} - k_{q,i,m} z_{i,m}^{q + 1} \\ & \quad + \Delta_{i,m - 1} + \eta_{1,i,m} + \eta_{2,i,m} + \sigma_{p,i,m} \theta_{i,m}^{p + 1} + \sigma_{q,i,m} \theta_{i,m}^{q + 1} \\ & \quad + z_{i,m} g_{i,m} z_{i,m} \\ & \le g_{i,m} z_{i,m} z_{i,m + 1} + \Sigma_{i,m} + \Delta_{i,m} \\ \end{aligned}$$

where

36 $$\begin{aligned} \Sigma_{i,m} & = \Sigma_{i,m - 1} - \rho_{p,i,m} \tilde{\theta }_{i,m}^{p + 1} - \rho_{q,i,m} \tilde{\theta }_{i,m}^{q + 1} - k_{p,i,m} z_{i,m}^{p + 1} - k_{q,i,m} z_{i,m}^{q + 1} \\ \Delta_{i,m} & = \Delta_{i,m - 1} + \eta_{1,i,m} + \eta_{2,i,m} + \sigma_{p,i,m} \theta_{i,m}^{p + 1} + \sigma_{q,i,m} \theta_{i,m}^{q + 1} \\ \end{aligned}$$

Based on the Lyapunov functionals (24) and (31), we choose the Lyapunov candidate functional

37 $$V = \sum\limits_{j = 1}^{n} {\frac{{z_{i,j}^{2} }}{2}} + \sum\limits_{j = 1}^{n} {\frac{1}{{2\mu_{i,j} }}\tilde{\theta }_{i,j}^{2} } .$$

We choose the virtual control laws as (13) and (19), the distributed adaptive fixed-time law and control based on the fixed-time adaptive technique and backstepping; this technique takes the trajectory along the system, based on the lemma in^13^, and therefore, the following inequality can be obtained:

38 $$\dot{V} \le - k_{p} V^{{\frac{p + 1}{2}}} - k_{q} V^{{\frac{q + 1}{2}}} + \Delta ,$$

where

39 $$\begin{aligned} k_{p} & = \frac{{\min \left\{ {k_{p,i,j \in N} ,\rho_{p,i,j \in N} } \right\}}}{{\left( {\max \left\{ {\frac{1}{2},\frac{1}{{2\mu_{i,j \in N} }}} \right\}} \right)^{{\frac{p + 1}{2}}} }},k_{q} = \frac{{\left( {2n} \right)^{{\frac{1 - q}{2}}} \min \left\{ {k_{q,i,j \in N} ,\sigma_{q,i,j \in N} } \right\}}}{{\left( {\max \left\{ {\frac{1}{2},\frac{1}{{2\mu_{i,j \in N} }}} \right\}} \right)^{{\frac{q + 1}{2}}} }} \\ \Delta & = \Delta_{i,n - 1} + \eta_{1,i,n} + \eta_{2,i,n} + \sigma_{p,i,n} \theta_{i,n}^{p + 1} + \sigma_{q,i,n} \theta_{i,n}^{q + 1} \\ \end{aligned}$$

The settling time $T$ satisfies

40 $$T \le T_{\max } = \frac{{2^{{\frac{3 - p}{2}}} }}{{k_{p} \left( {1 - p} \right)}} + \frac{2}{{k_{q} \left( {q - 1} \right)}}.$$

Therefore, the error closed-loop system has practical fixed-time stability based on the lemma in^32^. □

#### Remark 3

For practical engineering control, finite-time stability is obviously more practical than infinite-time stability. However, there are some limitations to finite-time stability because the convergence time of the designed system depends on the initial state. Therefore, in this section, we introduce another method, the tracking control method of nonaffine nonlinear leader–follower multiagent systems based on fixed-time stability theory. The upper bound of settling time does not depend on the initial state and can be realized by only adjusting the controller parameters.

#### Remark 4

The main difficulty in studying practical fixed-time stability is the sufficient condition and settling time based on the Lyapunov stability theorem. The practical fixed-time stability lemma in^32^ is based on fixed-time stability theory, and the procedure can be divided into two parts. The first part is the transfer condition to the practical fixed-time stability condition; then, the system is stable, and the settling time can be obtained based on fixed-time stability theory. There exist some different fixed-time stability conditions, and therefore, the settling time of practical fixed-time stability is not unique.

#### Remark 5

**Figure 2:**
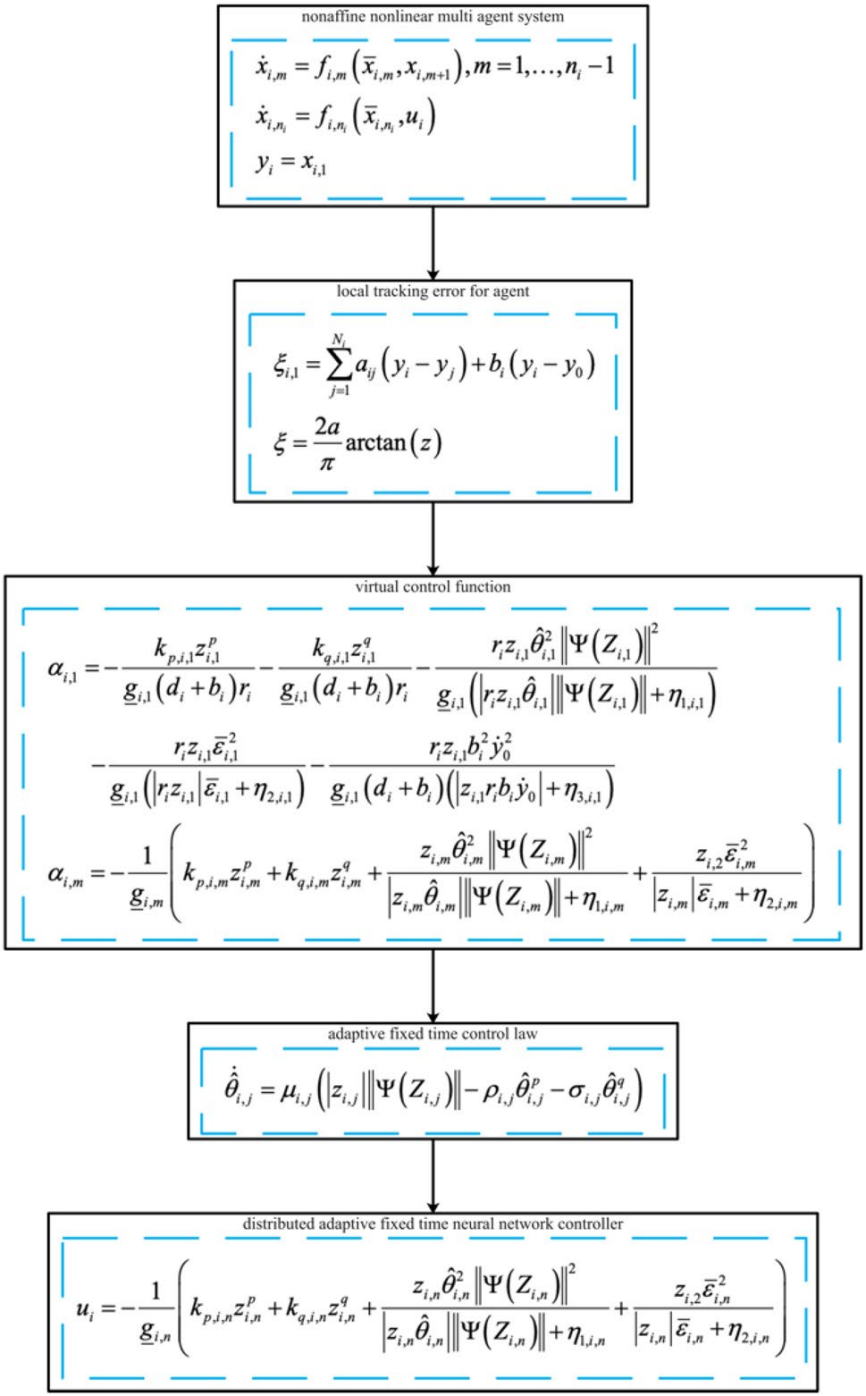
Design procedure.

The step-by-step design procedure is shown in Fig. 2.

Step 1: Design the ideal virtual control laws (13) and (19) based on the backstepping control technique.

Step 2: Design the distributed adaptive fixed-time control law (22) based on the fixed-time control theory.

Step 3: Obtain the actual controller (28) recursively through the virtual control signal and the adaptive parameter (22).

## Simulation study

Multiagent consensus control is widely used in practical industrial control, such as systems composed of multiple robots^50^ and multiple inverted pendulums^51^. In this section, an example (four robust follower agents and one lead agent) is presented to demonstrate the effectiveness of the proposed consensus control scheme for a nonaffine nonlinear multiagent system^48^. A step-by-step design procedure is shown to explain the control scheme.

**Figure 3:**
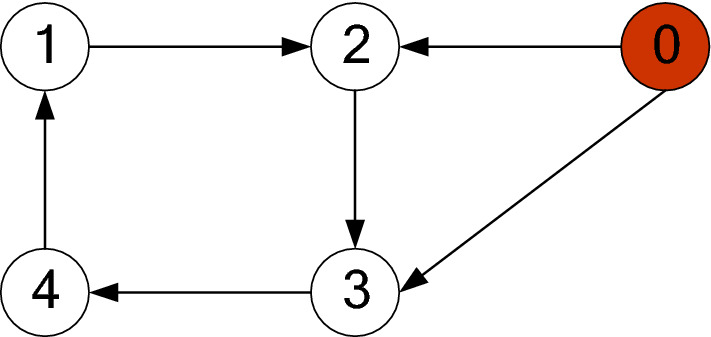
Topology of the multiagent system.

Consider the communication graph of the multiagent system in Fig. 3.

The leader agent is described as $5\sin \left( {0.1t} \right)$. The follower agents can be described as follows:

Agent 1:

41 $$\begin{aligned} \dot{x}_{1,1} & = 0.05\sin \left( {x_{1,1} } \right) + x_{1,2} + 0.1\sin \left( {x_{1,2} } \right) \\ \dot{x}_{1,2} & = 0.2 \times 4^{{ - x_{1,1}^{2} }} + \left( {54 + 3\exp \left( { - x_{1,1}^{2} } \right)} \right)u_{1} + 12\tanh \left( {u_{1} } \right) \\ \end{aligned}$$

Agent 2:

42 $$\begin{aligned} \dot{x}_{2,1} & = 0.1\sin \left( {x_{2,1} } \right) + x_{2,2} + 0.2\sin \left( {x_{2,2} } \right) \\ \dot{x}_{2,2} & = 0.2 \times 4^{{ - x_{2,1}^{2} }} + 20u_{2} + 2\cos \left( {u_{2} } \right) \\ \end{aligned}$$

Agent 3:

43 $$\dot{x}_{3,1} = u_{3} + 0.2u_{3} \exp \left( { - x_{3,1}^{2} } \right)$$

Agent 4:

44 $$\dot{x}_{4,1} = \sin \left( {x_{4,1} } \right)x_{4,1} + u_{4} + 0.1\sin \left( {u_{4} } \right)$$

Step 1: Design of the ideal virtual control laws based on the backstepping control technique.

45 $$\begin{aligned} \alpha_{i,1} & = - \frac{{k_{p,i,1} z_{i,1}^{p} }}{{\underline {g}_{i,1} \left( {d_{i} + b_{i} } \right)r_{i} }} - \frac{{k_{q,i,1} z_{i,1}^{q} }}{{\underline {g}_{i,1} \left( {d_{i} + b_{i} } \right)r_{i} }} - \frac{{r_{i} z_{i,1} \hat{\theta }_{i,1}^{2} \left\| {\Psi \left( {Z_{i,1} } \right)} \right\|^{2} }}{{\underline {g}_{i,1} \left( {\left| {r_{i} z_{i,1} \hat{\theta }_{i,1} } \right|\left\| {\Psi \left( {Z_{i,1} } \right)} \right\| + \eta_{1,i,1} } \right)}} \\ & \quad - \frac{{r_{i} z_{i,1} \overline{\varepsilon }_{i,1}^{2} }}{{\underline {g}_{i,1} \left( {\left| {r_{i} z_{i,1} } \right|\overline{\varepsilon }_{i,1} + \eta_{2,i,1} } \right)}} - \frac{{r_{i} z_{i,1} b_{i}^{2} \dot{y}_{0}^{2} }}{{\underline {g}_{i,1} \left( {d_{i} + b_{i} } \right)\left( {\left| {z_{i,1} r_{i} b_{i} \dot{y}_{0} } \right| + \eta_{3,i,1} } \right)}} \\ \end{aligned}$$

46 $$\alpha_{i,m} = - \frac{1}{{\underline {g}_{i,m} }}\left( {k_{p,i,m} z_{i,m}^{p} + k_{q,i,m} z_{i,m}^{q} + \frac{{z_{i,m} \hat{\theta }_{i,m}^{2} \left\| {\Psi \left( {Z_{i,m} } \right)} \right\|^{2} }}{{\left| {z_{i,m} \hat{\theta }_{i,m} } \right|\left\| {\Psi \left( {Z_{i,m} } \right)} \right\| + \eta_{1,i,m} }} + \frac{{z_{i,2} \overline{\varepsilon }_{i,m}^{2} }}{{\left| {z_{i,m} } \right|\overline{\varepsilon }_{i,m} + \eta_{2,i,m} }}} \right)$$

Step 2: Design of the distributed adaptive fixed-time control laws based on fixed-time control theory.

47 $$\dot{\hat{\theta }}_{i,j} = \mu_{i,j} \left( {\left| {z_{i,j} } \right|\left\| {\Psi \left( {Z_{i,j} } \right)} \right\| - \rho_{i,j} \hat{\theta }_{i,j}^{p} - \sigma_{i,j} \hat{\theta }_{i,j}^{q} } \right)$$

Step 3: Obtaining the actual controller recursively through the virtual control signal and the adaptive parameter.

48 $$u_{i} = - \frac{1}{{\underline {g}_{i,n} }}\left( {k_{p,i,n} z_{i,n}^{p} + k_{q,i,n} z_{i,n}^{q} + \frac{{z_{i,n} \hat{\theta }_{i,n}^{2} \left\| {\Psi \left( {Z_{i,n} } \right)} \right\|^{2} }}{{\left| {z_{i,n} \hat{\theta }_{i,n} } \right|\left\| {\Psi \left( {Z_{i,n} } \right)} \right\| + \eta_{1,i,n} }} + \frac{{z_{i,2} \overline{\varepsilon }_{i,n}^{2} }}{{\left| {z_{i,n} } \right|\overline{\varepsilon }_{i,n} + \eta_{2,i,n} }}} \right),$$

where $$\overline{z}_{11} = 4 \times 2^{ - t} + 0.2$$, $$\underline {z}_{12} = - 2^{ - 3t} - 0.2$$, $$\overline{z}_{21} = 4 \times 2^{ - t} + 0.2$$, $$\underline {z}_{22} = - 2^{ - 3t} - 0.2$$, $$\overline{z}_{31} = 4 \times 2^{ - t} + 0.2$$, $$\underline {z}_{32} = - 2^{ - 3t} - 0.2$$, $$\overline{z}_{41} = 4 \times 2^{ - t} + 0.2$$, and $$\underline {z}_{42} = - 2^{ - 3t} - 0.2$$.

The control parameters are designed as $p = \frac{1}{3},q = \frac{5}{3},k_{p,i,j} = k_{q,i,j} = 1$, and the upper bound of settling time is $T_{\max } = 6.7798$, which is calculated by (40).

**Figure 4:**
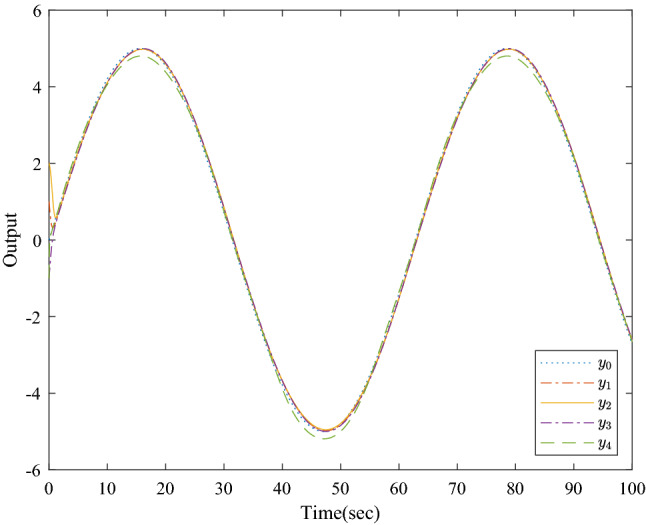
Output of five agents.

**Figure 5:**
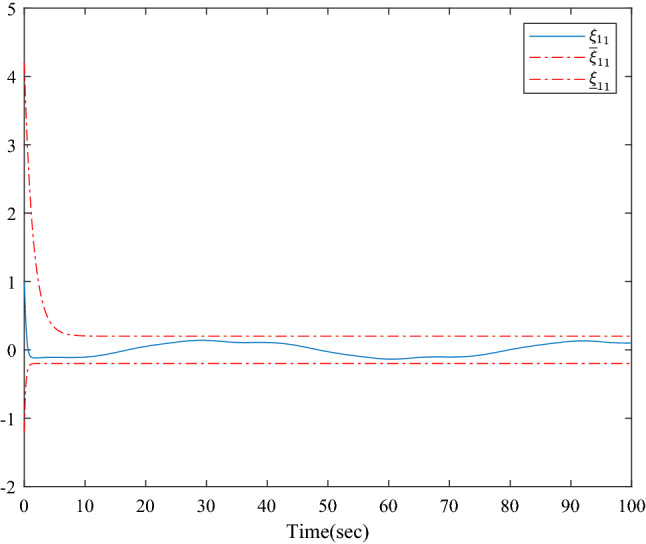
Error states of the following agents $\xi_{11}$ along with their bounds.

**Figure 6:**
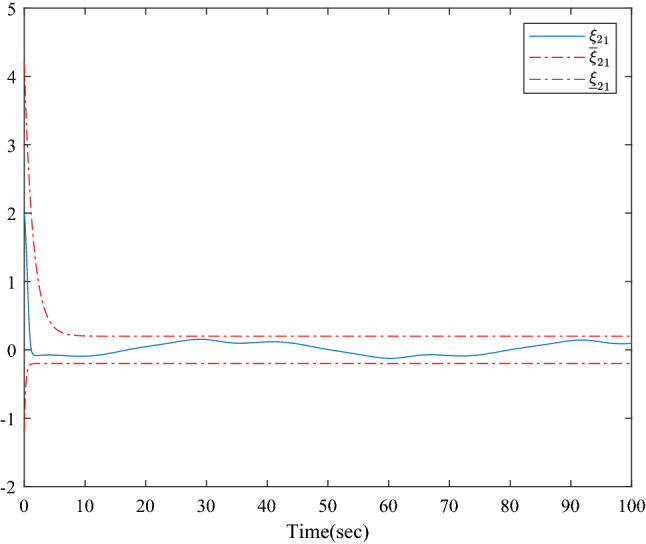
Error states of the following agents $\xi_{21}$ along with their bounds.

**Figure 7:**
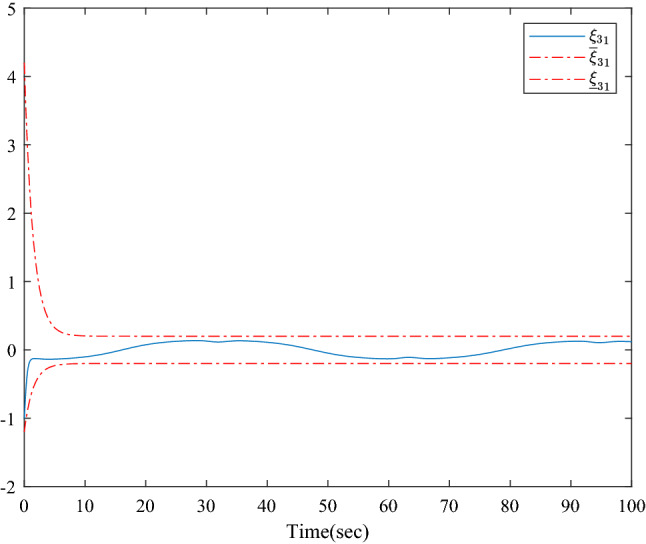
Error states of the following agents $\xi_{31}$ along with their bounds.

**Figure 8:**
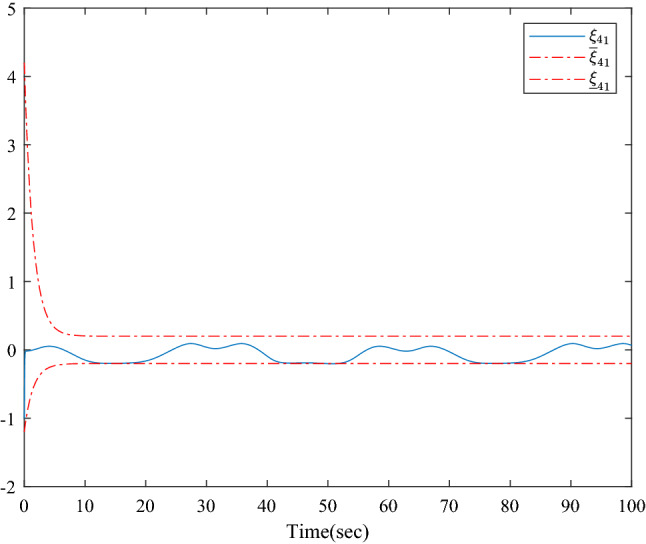
Error states of the following agents $\xi_{41}$ along with their bounds.

**Figure 9:**
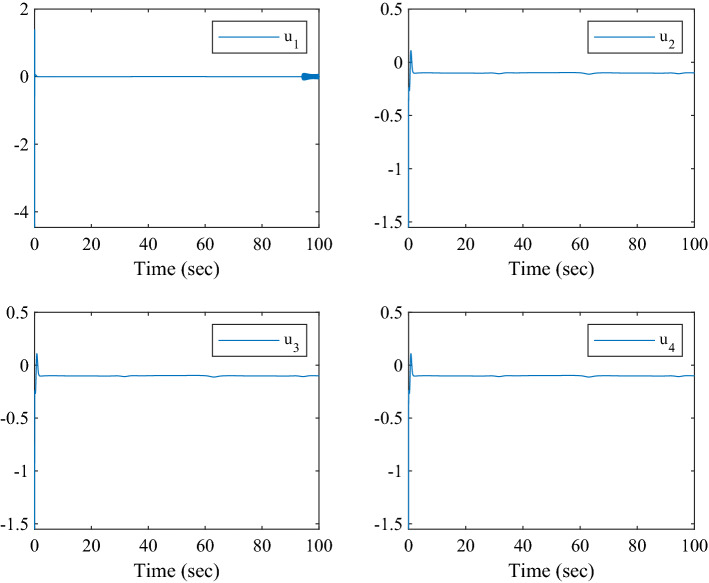
Distributed adaptive neural networks control laws of the multiagent system.

Figure 1 shows the consensus control structure of the closed error system. Figure 2 shows the step-by-step design procedure. Figure 3 is the communication graph of the multiagent system. Figures 4, 5, 6, 7, 8, 9 show the simulation results. The simulation results show that the follower agents can follow the leader agent in finite time and that the upper bound of settling time does not depend on the initial condition. Figure 4 shows the response curves of the outputs of the five agents based on the virtual control laws (45) and (46), and the neural networks adaptive controller (48), which indicate the performance of the distributed adaptive fixed-time neural networks controller. It should be noted that he reason for output $y_{4}$ of agent 4 is slightly off the reference trajectory $y_{0}$ of leader 0 is that the neural networks approximate nonlinear systems, and the error of approximation is appeared, but the error is converged to a small neighborhood rather than the origin point. Based on Lyapunov stability theorem, the error of the closed-loop system is practically fixed-time stable. Figures 5, 6, 7, 8 show the tracking errors between the state and the reference signal along with their bounds, which indicate that the local consensus error is bounded in all processes based on homeomorphism mapping technology. It can be observed that all the follower agents can follow the leader agent in a fixed time. Figures 5, 6, 7, 8 demonstrate that the tracking error of the system reaches consensus in fixed time and remains within the bounds. Figure 9 shows the curve of the distributed adaptive neural networks controller, which is bounded and reliable. From the simulation data, it can be calculated that the upper bound of the settling time is 6.7798 s. It can be obtained that consensus can be achieved in finite time. Therefore, the effectiveness of the proposed scheme can be illustrated. Compare with result in^47^, the advantage of fixed-time control design bound of the settling time, and the disadvantage is complex algorithm of controller.

## Conclusions

This article develops a fixed-time adaptive neural networks tracking control scheme to provide a new procedure for dealing with leader–follower multiagent consensus control systems. A simulation demonstrates the proposed scheme. There are several conclusive points, as summarized below.

This article focuses on consensus controller design for nonaffine nonlinear leader–follower multiagent systems. The controller is designed based on the neural networks technique. A fixed-time adaptive algorithm is presented for approximating the parameters of the neural networks. The fixed-time consensus analysis of error closed-loop systems is demonstrated based on Lyapunov fixed-time stability theory. The upper bound of settling time is independent from the initial parameters. The scheme proposed in this article is not limited to a nonaffine nonlinear leader–follower multiagent system. Furthermore, a step-by-step procedure is listed, which can be used by engineers to take up the proposed consensus control method with a computer for practical engineering tasks. Compared with previous research, the fixed-time neural networks adaptive control has potential for further expansion. A similar scheme can be constructed for high-order nonlinear multiagent systems.
